# Odorant Binding Proteins of the Desert Locust *Schistocerca gregaria* (Orthoptera, Acrididae): Topographic Expression Patterns in the Antennae

**DOI:** 10.3389/fphys.2018.00417

**Published:** 2018-04-17

**Authors:** Xingcong Jiang, Miriam Ryl, Jürgen Krieger, Heinz Breer, Pablo Pregitzer

**Affiliations:** ^1^Institute of Physiology, University of Hohenheim, Stuttgart, Germany; ^2^Department of Animal Physiology, Institute of Biology/Zoology, Martin Luther University Halle-Wittenberg, Halle, Germany

**Keywords:** locust, *Schistocerca gregaria*, odorant binding protein, sensilla, topographic expression

## Abstract

Odorant binding proteins (OBPs) enriched in the sensillum lymph are instrumental in facilitating the transfer of odorous molecules to the responsive receptors. In Orthopteran locust species, an in-depth understanding of this important soluble protein family is still elusive. In a previous study, we have demonstrated that the repertoire of locust OBPs can be divided into four major clades (I–IV) on the phylogenetic scale and for representatives of subfamily I-A and II-A a distinct sensilla-specific expression pattern was determined. In this study, by focusing on a representative locust species, the desert locust *Schistocerca gregaria*, we have explored the antennal topographic expression for representative OBPs of other subfamilies. First, subtypes of subfamily III-A and III-B were exclusively found in sensilla chaetica. Then, a similar expression pattern in this sensillum type was observed for subfamily I-B subtypes, but with a distinct OBP that was expressed in sensilla coeloconica additionally. Moreover, the atypical OBP subtype from subfamily IV-A was expressed in a subpopulation of sensilla coeloconica. Last, the plus-C type-B OBP subtype from subfamily IV-B seems to be associated with all four antennal sensillum types. These results profile diversified sensilla-specific expression patterns of the desert locust OBPs from different subfamilies and complex co-localization phenotypes of distinct OBP subtypes in defined sensilla, which provide informative clues concerning their possible functional mode as well as a potential interplay among OBP partners within a sensillum.

## Introduction

Insects utilize hair-like cuticle appendages, so called sensilla, to receive environmental olfactory signals (Steinbrecht, 1996; Hansson and Stensmyr, 2011; Suh et al., 2014). Hydrophobic odorous molecules have to travel through the aqueous sensillum lymph before reaching the receptors residing in the chemosensory membrane of olfactory neurons in the antennae (Vogt et al., 1999; Leal, 2013; Suh et al., 2014). This passage is supposed to be facilitated by odorant binding proteins (OBPs) in the sensillum lymph, an important soluble protein family that is capable to accommodate and transfer odorant molecules (Vogt and Riddiford, 1981; Pelosi et al., 2006, 2014; Vieira and Rozas, 2011). OBPs are short polypeptides of approximately 110–200 amino acids that fold into a globular shape forming an interior binding cavity, where the interaction with odorous molecules takes place (Sandler et al., 2000; Tegoni et al., 2004). The sequence of classic OBPs is characterized by six conserved cysteine (C) residues, a hall mark of classic OBPs; plus-C or minus-C OBPs are categorized with more or less than six conserved C-residues (Xu et al., 2003; Zhou et al., 2004; Foret and Maleszka, 2006; Vieira and Rozas, 2011). OBPs are produced by auxiliary cells which envelope the sensory neurons by their extended processes. The enrichment of OBPs in the sensillum types that respond to olfactory cues has been reported for many insect species (Pelosi et al., 2014, 2017). Beyond the olfactory sensilla, OBP expression has also been found in the sensilla that are seemingly dedicated to gustatory cues (Galindo and Smith, 2001; Jeong et al., 2013). Incidentally, besides the sensilla-specific expression in the chemosensory organs, like the antennae, OBPs are also expressed in other tissues of which the functional connotations seem to be less associated with chemical communication (Pelosi et al., 2017).

*Schistocerca gregaria*, the desert locust, represents a model organism of the Orthopteran order, which emerged much earlier than the Lepidopteran and Dipteran orders on the evolutionary scale (Wheeler et al., 2001; Vogt et al., 2015). Locusts are characterized by a hemimetabolous life circle and a population density dependent behavioral plasticity, which involves the perception of behavioral relevant semiochemicals (Pener and Yerushalmi, 1998; Hassanali et al., 2005; Guo et al., 2011; Wang and Kang, 2014). For locust species an in-depth understanding of the OBP family from either molecular or cellular perspective is still elusive (Ban et al., 2003; Jin et al., 2005; Jiang et al., 2009; Xu et al., 2009; Yu et al., 2009). Previously, we have conducted a comprehensive sequence analysis of the OBP families from *Schistocerca gregaria* and three other locust species which classifies locust OBPs into several categories, e.g., classic, plus-C type-A, plus-C type-B, minus-C and atypical OBPs. Based on the phylogenetic relationship locust OBPs reside within four major phylogenetic clades. Concentrating on the two OBP subfamilies I-A and II-A, which comprise the classic OBP subtypes, we have found a characteristic sensilla-specific expression pattern for the desert locust OBP representatives in the antennae (Jiang et al., 2017). In the present study, we set out to explore the antennal topographic expression of desert locust OBPs from the remaining subfamilies on the phylogenetic tree.

## Materials and Methods

### Animals and Tissue Collection

The desert locust *Schistocerca gregaria* reared on the gregarious phase were purchased from Bugs-International GmbH (Irsingen/Unterfeld, Germany). Antennae of adult male and adult female were dissected using autoclaved surgical scissors and were immediately frozen in liquid nitrogen. Tissues were stored at -70°C before subsequent RNA extraction.

### RNA Extraction and Reverse Transcription PCR (RT-PCR)

Total RNA was extracted from the frozen tissues using TRIzol reagent (Invitrogen) following the protocol recommended by the manufacturer. The poly (A)^+^ RNA was purified from 100 μg of total RNA using oligo (dT)_25_ magnetic dynabeads (Invitrogen) conforming to the recommendation of the supplier. The generated mRNA was reverse transcribed to cDNA in a total volume of 20 μl employing SuperScript^TM^ III Reverse Transcriptase (Invitrogen). PCR conditions used in RT-PCR experiments were: 94°C for 1 min 40 s, then 20 cycles with 94°C for 30 s, 60°C for 30 s and 72°C for 2 min, with a reduction in the annealing temperature by 0.5°C per cycle, which was followed by a further cycles (20 times) on the condition of the last cycling step (annealing temperature was 50°C) and a final extension step for 7 min at 72°C. The sense (s) and antisense (as) primer pairs used for amplification of the desert locust OBP coding sequences were:

OBP2 s, atggccagccattgccacgccacc
OBP2 as, ttctccggatttcctaaactccgc
OBP3 s, atgctgctggcagcccccgcaaagg
OBP3 as, ctttttcctgatcaagcatccacc
OBP4 s, cctgtggcgacacttggtggccg
OBP4 as, gcctttagccatcatcccctt
OBP7 s, cgatgtgcttcgtcggtgggtgat
OBP7 as, acgtcgttctcgtcggactctgga
OBP8 s, agactcgccaacccgccaca
OBP8 as, ttctgacggggcgtgtggga
OBP9 s, gccacagtccggtgcagcat
OBP9 as, aatctggtcgctgacgcact
OBP12 s, acaactcttgcagccatgaagtgg
OBP12 as, tccacttcttgttcccatactggt
OBP13 s, gagctgaggtaatgaagagggtca
OBP13 as, cctgcacattcagatccaagcagc

The primer pairs against other desert locust OBP subtypes were given in (Jiang et al., 2017).

### Synthesis of Riboprobes for *in Situ* Hybridization

PCR products of the desert locust OBP coding sequences were sequenced and then cloned into pGEM-T vectors (Invitrogen) for the subsequent *in vitro* transcription. The linearized pGEM-T vectors consisting of desert locust OBP coding sequences were utilized to synthesize both sense and antisense riboprobes labeled with digoxigenin (Dig) or biotin (Bio) using the T7/SP6 RNA transcription system (Roche, Germany). The synthesis procedure stringently followed the protocol provided by the manufacturer.

### *In Situ* Hybridization

Antennae of adult *Schistocerca gregaria* were dissected and embedded in Tissue-Tek O.C.T. Compound (Sakura Finetek Europe, Netherlands). Cryosections with a 12 μm-thickness were thaw mounted on SuperFrost Plus slides (Menzel-Gläser, Braunschweig, Germany) at -21°C (Jung CM300 cryostat). RNA *In situ* hybridization was performed as previously reported (Yang et al., 2012; Guo et al., 2013; Jiang et al., 2016, 2017). In brief, the cryosections were firstly fixed (4% paraformaldehyde in 0.1 M NaHCO_3_, pH 9.5) at 4°C for 22 min, followed by a series of treatments at room temperature: a wash for 1 min in PBS (phosphate buffered saline = 0.85% NaCl, 1.4 mM KH_2_PO_4_, 8 mM Na_2_HPO_4_, pH 7.1), an incubation for 10 min in 0.2 M HCl, another wash for 1 min in PBS, an incubation for 10 min in acetylation solution (0.25% acetic anhydride freshly added in 0.1 M triethanolamine) and washes for three times in PBS (3 min each). Afterward, the sections were pre-hybridized for 1 h at 60°C bathed in hybridization buffer (50% formamide, 5x SSC, 50 μg/ml heparin, and 0.1% Tween-20). A volume of 150 μl hybridization solution containing experiment riboprobes in hybridization buffer was evenly applied onto the tissue section. A coverslip was placed on top and slides were incubated in a moister box at 60°C overnight (18–20 h). After hybridization, slides were washed twice for 30 min in 0.1x SSC at 60°C, then each slide was treated with 1 ml 1% blocking reagent (Roche) for 35 min at room temperature.

Visualization of Dig-labeled riboprobe hybridizations was achieved by using an anti-Dig alkaline phosphatase (AP) conjugated antibody (1:500, Roche) and NBT/BCIP as substrates. Antennal sections were analyzed on a Zeiss Axioskope2 microscope (Zeiss, Oberkochen, Germany) equipped with Axiovision software. For two-color fluorescent *in situ* hybridization visualization of hybridized riboprobes was performed by using an anti-Dig AP-conjugated antibody in combination with HNPP/Fast Red (Roche) for Dig-labeled probes and an streptavidin horse radish peroxidase-conjugate together with fluorescein-tyramides as substrate (TSA kit, Perkin Elmer, Waltham, MA, United States) for biotin-labeled probes. Tissue sections in two-color FISH experiments were analyzed with a Zeiss LSM510 Meta laser scanning microscope (Zeiss, Oberkochen, Germany), and the acquired confocal images stacks were processed by ZEN 2009 software. The images presented in this paper integrate the projections of a series of optical planes selected from continuous confocal image stacks. For clear data presentation, images were only adjusted in brightness and contrast. It is noted that the images obtained via the two-color FISH approach always contained the cuticle unspecifically stained, most likely due to the intrinsic fluorescence. To clarify the specific fluorescent labeling, a dashed line was added to indicate the interface between the cuticle and the cellular layers. Antennal sections of both male and female were analyzed under the same experimental conditions and were tested with each generated riboprobes. There were no discernible gender dependent differences regarding to the labeling intensity as well as the labeling pattern. Therefore, only the images acquired from male antenna sections were presented in this paper.

## Results

### Topographic Expression Patterns of OBP Subtypes From Clade I and III

**FIGURE 1:**
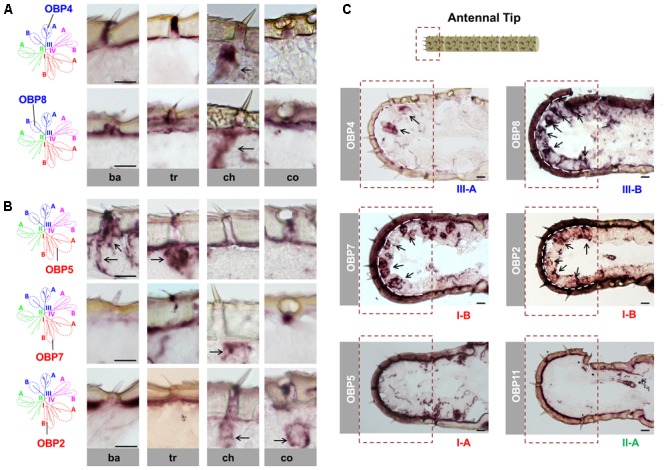
Sensilla chaetica express OBP subtypes of two phylogenetic clades. The schematic diagram of the phylogenetic tree (left in **A,B**) was adapted from Jiang et al. (2017) where OBP families of four locust species have been analyzed. The specific *S. gregaria* OBP subtypes studied in this analysis were indicated. A detail classification of different subfamilies is illustrated in **Supplementary Figure S1**. Topographic expression of OBPs was visualized by using antisense riboprobes specifically targeting distinct OBP subtypes in conjunction with chromogenic *in situ* hybridization (ISH). **(A,B)** Visualization of the labeled cells expressing distinct OBP subtypes of subfamily III-A, III-B, I-A, and I-B in four morphological types of antennal sensilla. Ba, sensilla basiconica; Tr, sensilla trichodea; Ch, sensilla chaetica; Co, sensilla coeloconica. The visible labeled structures are denoted by black arrows. **(C)** Visualization of the cells expressing distinct OBP subtypes from different subfamilies on the tip of the antennae. Notably, sensilla chaetica are exclusively enriched on the antennal tip (Ochieng et al., 1998). The area of the antennal tip is indicated by a box with a dashed line. The visible cell clusters are denoted by black arrows, and in some images the interface between the cuticle and cellular layer is depicted as a white dashed line. The subfamily to which a distinct OBP subtype belongs is annotated below the images. Scale bars, 20 μm.

A previously performed phylogenetic analysis of OBPs from four locust species revealed that the locust OBP family can be divided into four major clades consisting of three conserved subfamilies. For the two subfamilies I-A and II-A, which both comprise classic OBP subtypes, we found that the representative I-A subtypes are expressed in sensilla basiconica and sensilla trichodea, whereas the representative II-A subtypes are expressed in sensilla coeloconica (Jiang et al., 2017). In this study, we concentrated on the conserved subfamily III-A, which includes the plus-C type-A OBP subtypes that share only low sequence identities with the classic OBP subtypes. In order to explore their sensilla-specific expression pattern, we adopted the strategy of mRNA *in situ* hybridization and assessed the expression of OBP4, a representative subtype of subfamily III-A, in the four morphologically distinguishable types of antennal sensilla. The results of these approaches revealed a discernible labeling of OBP4 expressing cells in sensilla chaetica; no labeling was visible in any of the other three sensillum types (**Figure 1A**). Apart from the subfamily III-A, clade III also comprises subfamily III-B, which includes the classic OBP subtype OBP8 and its orthologs. Analysis of the expression pattern revealed that OBP8-positive cells were also exclusively enriched in sensilla chaetica, thus resembling the plus-C type-A subtype OBP4 (**Figure 1A**). Together, these results imply that OBP subtypes of the clade III are specifically expressed in sensilla chaetica and thus deviate from the distribution of OBP subtypes from subfamilies I-A and II-A (Jiang et al., 2017).

In view of a clade-specific spatial expression pattern as seen for clade III (see above) it is interesting to note that clade I comprises, besides the conserved subfamily I-A, the more divergent subfamily I-B (**Supplementary Figure S1**). Since representatives of subfamily I-A were found to be restricted to sensilla basiconica and trichodea (**Figure 1B**) (Jiang et al., 2017), the question arises, whether OBPs of subfamily I-B may also be expressed in the same sensillum types. To scrutinize this notion, we have analyzed OBP2 and OBP7, the two subtypes in subfamily I-B. The results are depicted in **Figure 1B** and indicate that labeling for OBP2 and OBP7 was neither found in sensilla basiconica nor in sensilla trichodea; however, the labeling was present in sensilla chaetica and for OBP2 the labeled cells were concomitantly visible in sensilla coeloconica (**Figure 1B**). These data indicate that the topographic distribution of subfamily I-B OBPs clearly deviate from that of their counterparts of subfamily I-A and demonstrate that there is no clade-specific spatial expression pattern for members of clade I.

Previous anatomical studies have shown that sensilla chaetica are highly enriched at the tip of the antennae, a region with relatively few of the other three sensillum types (Ochieng et al., 1998). This spatial segregation of sensilla chaetica allows a more detailed analysis of the four identified OBP subtypes in this sensillum type. As shown in **Figure 1C**, numerous labeled cells were visualized using the probes for OBP4 (subfamily III-A), OBP8 (subfamily III-B) as well as OBP2 and OBP7 (subfamily I-B). In contrast, with the riboprobes for OBP subtypes that are specifically expressed in other sensillum types, such as OBP5 (subfamily I-A) and OBP11 (subfamily II-A), no discernible labeling was found at the antennal tip (**Figure 1C**).

### Co-localization of OBP Subtypes From Different Subfamilies in Sensilla Chaetica

**FIGURE 2:**
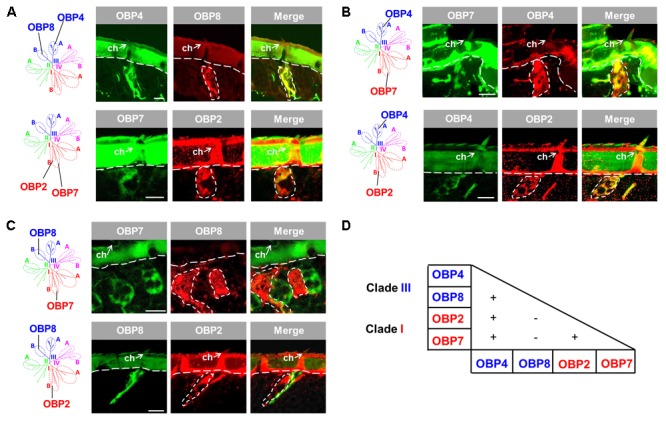
Co-localization of four OBP subtypes from two clades in sensilla chaetica. The relative localization of OBP types was analyzed by two-color fluorescent *in situ* hybridization (FISH) using combinations of specific DIG- or biotin-labeled antisense riboprobes against distinct OBP subtypes. **(A)** OBP subtypes of the same phylogenetic clade are co-expressed in the same set of cells in sensilla chaetica (ch). OBP4 and OBP8 belong to clade III, and OBP2 and OBP7 belong to clade I. **(B)** OBP2 and OBP7 residing in subfamily I-B are co-expressed with OBP4 from subfamily III-A in the same set of cells in sensilla chaetica. **(C)** OBP2 and OBP7 residing in subfamily I-B are expressed in a different set of cells from OBP8 (subfamily III-B). It is noted that the labeling for OBP7 cells pronounces a distinct cell population in a sensillum chaeticum different from the one containing OBP8 expressing cells. In contrast, OBP2 and OBP8 labeled cells were found in the same sensillum chaeticum. The interface between the cuticle and cellular layer is depicted by a white dashed line. Distinct cell clusters visualized by the DIG-labeled probes (red) are encircled by white dashed lines. These areas are indicated also on the images showing the merged red and green fluorescence channels. **(D)** Recapitulation of the co-localization relationship among the four sensilla chaetica-positive OBP subtypes. The expression of two OBP subtypes in the same set of cells is denoted as “+”, while “–” indicates expression of two OBP subtypes in different set of cells. The color code to distinguish OBP subtypes conforms to that for the phylogenetic analysis (**Supplementary Figure S1**). Scale bars, 20 μm.

Since the four OBP subtypes reside in two different phylogenetic clades, we ask whether the different OBP subtypes are present in the same set of cells or in distinct cell populations of sensilla chaetica. To approach this question, we have generated either DIG- or BIO-labeled riboprobes for each OBP subtype and by means of two-color FISH analysis we have visualized the relative topographic localization of the labeled cells (**Figure 2**). In a first step, we have analyzed the subtypes from the same phylogenetic clade. For the two subtypes from clade III, OBP4 and OBP8, a widely overlapped labeling was found indicating that they were co-localized in the same set of cells in many, if not all, inspected sensilla chaetica (**Figure 2A**). Analysis for the two subtypes from subfamily I-B, OBP2 and OBP7, also revealed a largely overlapped labeling (**Figure 2A**). These results suggest that within clade III and subfamily I-B OBP subtypes are generally expressed in the same set of cells in sensilla chaetica. In a next step, we explored whether OBP subtypes from different clades may either be expressed in the same or a different set of cells. For the member of subfamily III-A (OBP4) and the members of subfamily I-B (OBP2 and OBP7) a largely overlapping labeling was observed (**Figure 2B**). However, for the member of subfamily III-B (OBP8) and the members of subfamily I-B (OBP2 and OBP7) no labeling overlap was found (**Figure 2C**). While labeling for OBP2 and OBP8 was found in different sets of cells of the same sensillum chaeticum, interestingly, OBP7 seemed to be present in the cells of distinct sensilla chaetica which differ from sensilla with OBP8-positive cells (**Figure 2C**). These results emphasize the complex co-localization relationship among OBP2, OBP4, and OBP8. The notion that OBP4 and OBP8 may be separately expressed in a subset of sensilla chaetica was confirmed upon a comprehensive inspection of the labeling for OBP4 and OBP8 (**Supplementary Figure S2**), indicating a broader expression scope for OBP4 in certain sensilla chaetica. In sum, the results indicate that sensilla chaetica express OBP subtypes from more than one phylogenetic clade, and co-localization of the OBP subtypes in distinct sensilla subtypes occurs in a combinatorial mode.

### OBP2, Member of Subfamily I-B, Is Expressed in Sensilla Coeloconica and Chaetica

**FIGURE 3:**
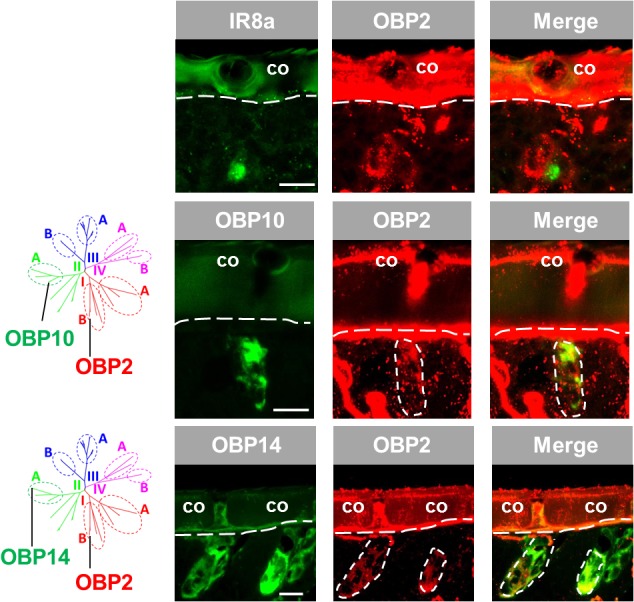
OBP2 from subfamily I-B is expressed in sensilla coeloconica and sensilla chaetica. The relative localization of OBP2 and the marker genes indicating expression in sensilla coeloconica (co) was analyzed by utilizing antisense riboprobes targeting specific molecular elements in conjunction with two-color FISH. (Upper) OBP2 expressing cells surround a sensory neuron positive for IR8a, a specific molecular marker for sensilla coeloconica. (Middle and lower) OBP10 and OBP14 from the subfamily II-A are specifically expressed in sensilla coeloconica and are employed to mark two different sets of auxiliary cells in this sensillum type (Jiang et al., 2017). The interface between the cuticle and the cellular layer is denoted by a white dashed line. Distinct cell clusters positive for the DIG-labeled OBP2 probe (red) are encircled by white dashed lines. The position of these cell clusters is also indicated on the images showing the merged red and green fluorescence channels. Scale bars, 20 μm.

The results depicted in **Figure 1** indicate that OBP2, a subtype of subfamily I-B, may not only be expressed in sensilla chaetica (see above) but also in sensilla coeloconica. To substantiate the observation that OBP2 is in fact expressed in sensilla coeloconica, we utilized IR8a, the co-receptor of divergent IRs (Abuin et al., 2011; Guo et al., 2013), as a specific marker of sensory neurons housed in sensilla coeloconica. The results of double labeling experiments indicate that labeled OBP2 cells are tightly surrounding IR8a-positive cells in sensilla coeloconica (**Figure 3**). Given that in sensilla coeloconica OBP subtypes of subfamily II-A are specifically expressed, the question arises as to whether OBP2, a member of subfamily I-B, may be co-expressed with OBP subtypes of subfamily II-A. As representatives for subfamily II-A OBP10 and OBP14 were investigated. The results depicted in **Figure 3** indicate that the labeling for OBP2 indeed overlapped with that for the subfamily II-A representatives, indicating that in a set of sensilla coeloconica OBP subtypes from subfamily I-B and subfamily II-A coexist. Furthermore, the results confirm that OBP2 is in fact present in the two types of sensilla, sensilla coeloconica and sensilla chaetica.

### Topographic Expression Pattern of an Atypical OBP Subtype From Subfamily IV-A

**FIGURE 4:**
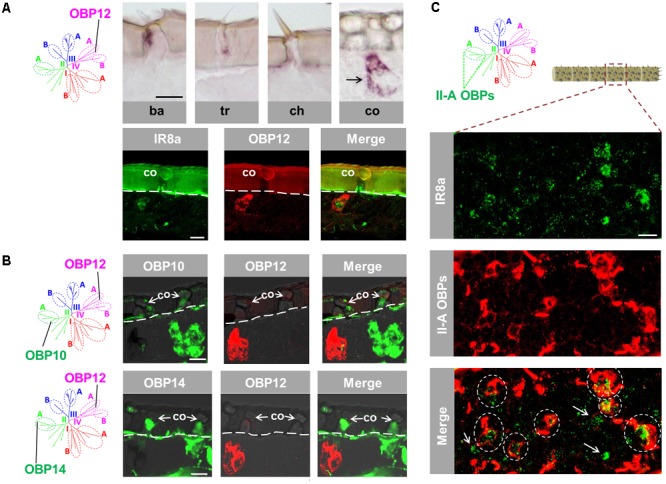
An atypical OBP subtype pronounces a segregated subpopulation of sensilla coeloconica. **(A)** OBP12, an atypical OBP subtype residing in subfamily IV-A, is exclusively expressed in sensilla coeloconica (co). Upper panel: OBP12 expressing cells were analyzed in four morphological types of antennal sensilla using specific riboprobe by means of ISH. Labeled OBP12 cells were detected only in sensilla coeloconica and are indicated by a black arrow. Ba, sensilla basiconica; Tr, sensilla trichodea; Ch, sensilla chaetica; Co, sensilla coeloconica. Lower panel: A co-localization of OBP12 expressing cells and an IR8a-positive neuron in sensilla coeloconica was visualized by means of two-color FISH. **(B)** The labeling of OBP12-positive cells does not overlap with the labeling of cells expressing OBP10 and OBP14 from subfamily II-A. The interface between the cuticle and the cellular layer is depicted by a white dashed line. **(C)** Three OBP subtypes of subfamily II-A label the major population of auxiliary cells in sensilla coeloconica. The presented optical view was adopted from a distal antennal segment and presumably illustrates the typical association between IR8a neurons and subfamily II-A OBP cells. The utilized DIG-labeled probes representing the three ortholog groups comprised in subfamily II-A (**Supplementary Figure S1**) were generated by mixing the riboprobes against OBP10, OBP11, and OBP14, respectively, at a ratio of 1:1:1. Areas encircled by white dashed lines indicate IR8a neurons that are co-localized with auxiliary cells expressing the subfamily II-A OBPs in the same coeloconic sensillum. White arrows indicate those IR8a neurons that are presumably not associated with auxiliary cells expressing subfamily II-A OBPs. Scale bars, 20 μm.

The atypical OBP subtypes converge onto the subfamily IV-A (**Supplementary Figure S1**) and are characterized by an extraordinary long span between C1 and C2 in comparison to the classic OBP subtypes (Jiang et al., 2017). This unique feature has raised the question whether atypical OBP subtypes may be expressed in specific sensillum types and/or in distinct cell populations. To approach this question, we have analyzed the expression pattern of OBP12, a subtype of subfamily IV-A. The results of labeling experiments are depicted in **Figure 4A** and indicate that OBP12 expressing cells were exclusively located in sensilla coeloconica. The sensilla specificity was subsequently confirmed by demonstrating the co-localization of OBP12 expressing cells and IR8a-positive cells in one sensillum coeloconicum (**Figure 4A**). Since OBPs of subfamily II-A are specifically expressed in sensilla coeloconica, we explored whether OBP12 may be co-localized with OBPs of subfamily II-A. Intriguingly, we found that the labeling for OBP12 cells did not overlap with the cells positive for OBP10 or OBP14 (**Figure 4B**), suggesting that OBP12 is expressed in a distinct subset of sensilla coeloconica.

It is yet unclear how many IR8a-positive neurons are surrounded by the auxiliary cells that express OBPs of subfamily II-A. To scrutinize this notion, double labeling experiments were performed with a probe for IR8a and a mix of riboprobes for OBP10, OBP11 and OBP14, which represent the three ortholog groups in subfamily II-A (**Supplementary Figure S1**). The results depicted in **Figure 4C** indicate that a considerable portion of IR8a-positive cells are engulfed by cells expressing OBPs of subfamily II-A (ovals in dash line). The remaining fraction of IR8a neurons seems to express non-II-A OBP subtypes, possibly OBP12. Together the results indicate that the atypical OBP subtype OBP12 is expressed in a segregated population of sensilla coeloconica.

### Topographic Expression and Sensillum-Association of a Plus-C Type-B OBP Subtype

**FIGURE 5:**
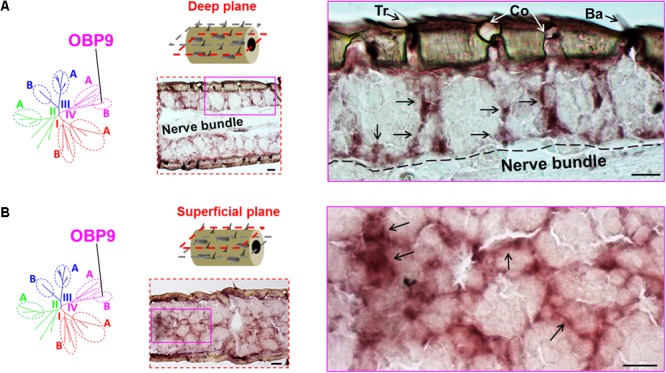
Topographic expression of the plus-C type-B OBP9 in the antennae. The topographic expression of OBP9 was analyzed by using a specific antisense riboprobe in conjunction with ISH. **(A,B)** Labeling of OBP9 expressing cells in two different anatomical planes of the antennae. OBP9 represents the plus-C type-B OBPs that are grouped into subfamily IV-B (diagrams, left lane). Two different horizontal planes are shown to visualize the OBP9 expression pattern: the first deep plane (**A**, middle lane, red dashed frame) penetrates into the central nerve bundle; the second superficial plane (**B**, middle lane, red dashed frame) is located between the cuticle and central nerve bundle. For each plane a selected area (magenta box, middle lane) of the analyzed section is shown at a higher magnification on the right. Black arrows indicate the visible cell bodies as well as their extended processes. The border between the cellular layer and the nerve bundle is depicted by a black dashed line. Tr, sensilla trichodea; Co, sensilla coeloconica; Ba, sensilla basiconica. Scale bars, 20 μm.

We have previously distinguished two categories of the plus-C OBPs based on the distinct conserved-C-patterns (Jiang et al., 2017). While the type-A OBP subtypes are grouped into the subfamily III-A, the type-B OBP subtypes are grouped into the subfamily IV-B (**Supplementary Figure S1**). Whereas type-A OBPs are expressed in sensilla chaetica (**Figure 1**), the expression pattern of type-B OBP subtypes is unclear. It is possible that the type-B OBPs share the sensilla specificity either with their close relatives in subfamily IV-A, e.g., OBP12, or with their type-A counterparts in subfamily III-A, e.g., OBP4. To approach this question, we have used a specific riboprobe for OBP9, a representative plus-C type-B subtype and assessed series of horizontal sections through the antennae. Upon an inspection of a deep anatomical plane close to the antennal nerve bundle, we found labeled structures for OBP9 which seemed to be less associated with a specific sensillum type, as typically found for the other OBP subtypes (**Figures 1, 3, 4**). Nevertheless, labeled cell bodies seemed to extend cytoplasmic processes which enclosed sensory neurons (**Figure 5A**). Interestingly, when we inspected an anatomical plane located closer to the cuticle, a more intense labeling was observed and a distinct nest-like labeling pattern for OBP9 emerged (**Figure 5B**).

**FIGURE 6:**
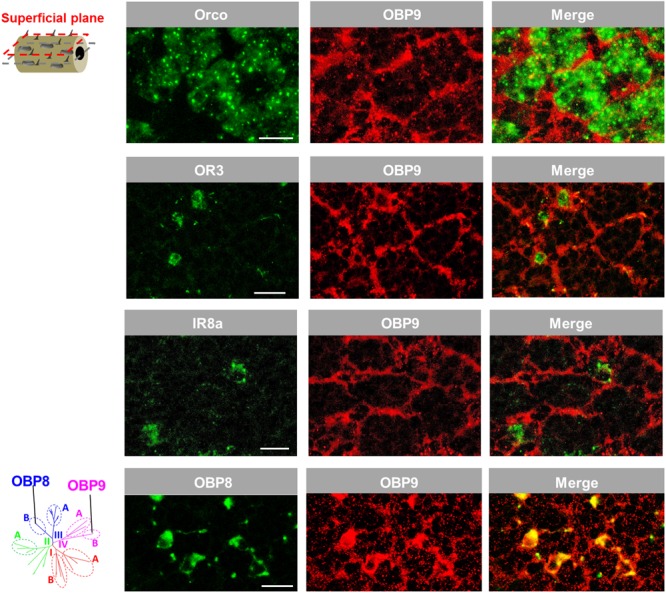
OBP9 expressing cells associate with four types of antennal sensilla. The relative localization of OBP9 and different marker genes indicative of specific sensillum types was analyzed by utilizing specific antisense riboprobes and the means of two-color FISH. Presented images were obtained from superficial cellular planes approaching the cuticle by performing series of horizontal sections of the antennae (diagram, left lane; similar to **Figure 5B**). Orco, OR3, and IR8a were utilized as the specific molecular markers of neurons housed in sensilla basiconica, sensilla trichodea, and sensilla coeloconica, respectively. OBP8 was used as a marker for auxiliary cells of sensilla chaetica (see **Figure 1**). Scale bars, 20 μm.

The notion that OBP9 labeling seems to be associated with multiple sensillum types was scrutinized by analyzing a possible co-localization of OBP9 labeling with markers for distinct neuron types. In a first approach, Orco, the obligate co-receptor of ORs, was used to label the multiple sensory neurons in sensilla basiconica (Ochieng et al., 1998). It was found that OBP9 cells tightly surrounded the Orco-positive neuron clusters (**Figure 6**). Similarly, OR3 was used as a marker for sensilla trichodea and IR8a was used as a marker for sensilla coeloconica; it was observed that OBP9 labeling engulfed OR3- and IR8a- expressing neurons (**Figure 6**). OBP8 is considered to be specific for sensilla chaetica (**Figure 1**) and the results of double labeling experiments with OBP9 and OBP8 clearly indicated a co-localization (**Figure 6**). Together, these results indicate an association of the plus-C type-B OBP9 with all four antennal sensillum types.

## Discussion

**FIGURE 7:**
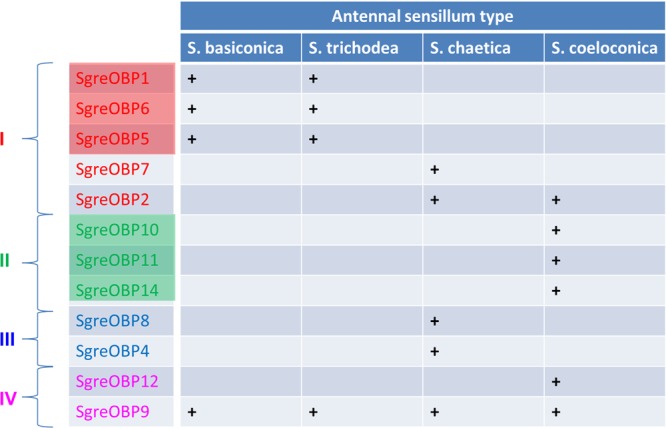
Antennal sensilla specificity of the desert locust OBP family. A distinct OBP subtype that is ascertained to be expressed in a specific sensillum type is denoted as “+”, whereas a blank field indicates the absence of particular OBP subtype in this sensillum type. The color code for individual OBPs subtypes is identical to the one used in the phylogenetic analysis (**Supplementary Figure S1**). Color shadings represent subfamily I-A and II-A, respectively.

Insects have evolved sensilla that are diversified in the external morphology as well as in the repertoire of molecular elements to act as versatile communication channels for environmental chemical signals (Hansson and Stensmyr, 2011; Leal, 2013; Suh et al., 2014). OBPs are considered to play an important role toward this task due to their capacity to accommodate and transfer odorous molecules. The present study, in conjunction with our previous work (Jiang et al., 2017), has concentrated on this important class of soluble proteins in the locust species *Schistocerca gregaria*, trying to decipher the principles how the multiple OBP subtypes are allocated among and within different sensillum types present on the locust antennae. The findings of this study revealed that subtypes of the desert locust OBP family display a diversified sensilla-specific expression profile and a complex co-localization phenotype in defined sensilla (**Figure 7**). Uncovering the sensillar and cellular organization pattern of distinct locust OBP subtypes may allow a first glimpse on their putative functional role as well as their potential interplay with distinct co-partners.

Our results indicate that several OBP subtypes from two phylogenetic clades are expressed in sensilla chaetica (**Figure 1**). A plus-C type-A subtype together with three classic subtypes were found to be co-expressed in a set of sensilla chaetica (**Figure 2**); this scenario is reminiscent of what was previously reported for sensilla trichodea of *Anopheles gambiae* (Schultze et al., 2013). Sensilla chaetica are characterized by distinct structural features, such as a thick and poreless cuticle wall, an apical pore and relatively few dendrites (Ochieng et al., 1998; Zhou et al., 2009); consequently, sensilla chaetica are considered as relevant for the reception of gustatory tastants rather than odorants. For the fruit fly this view was supported by extracellular recordings, calcium imaging and behavioral assays (Montell, 2009; Chen and Amrein, 2017; Scott, 2018). This view may also hold true for sensilla chaetica in locusts which are enriched on the tip of the antennae and palps (Blaney and Chapman, 1969; Ochieng et al., 1998) and are proposed with a receptive role of contact stimuli (Blaney, 1974, 1975; Saini et al., 1995). Thus, the presence of four OBP subtypes in sensilla chaetica on the tip of the antennae (**Figure 1**) suggests that these OBPs may be tuned to mediate the reception of gustatory stimuli. This view would be analogous to the finding for *Drosophila melanogaster* where OBP subtypes expressed in gustatory sensilla are involved in the reception of tastants (Jeong et al., 2013). This is further supported by a recent study demonstrating that knock-down of a sensilla chaetica-specific OBP subtype in *Locusta migratoria* caused a reduced neuronal response to chemical stimuli (Zhang et al., 2017). This finding further supports the notion that OBPs are intimately involved in detecting chemical compounds via sensilla chaetica. Intriguingly, it has been reported that the sensilla chaetica of locust, as well as contact sensilla of other insect species, have a sensillum lymph cavity which is separated into an inner and outer compartment (Ochieng et al., 1998; Shanbhag et al., 2001; Zhou et al., 2009). In a recent study, the labeling for an OBP subtype in *Locusta migratoria* was mainly observed in the non-innervated outer lumen, but not in the inner sensillum lymph which baths the chemosensory dendrites (Yu et al., 2009); this observation has led to speculations of how the cognitive ligands may reach the chemosensory dendrites. The discovery that four distinct OBP subtypes are expressed in this sensillum type (**Figures 1, 2**) opens the door for revisiting this aspect in more detail.

Distinct OBP subtypes from three phylogenetic clades were found to be expressed in sensilla coeloconica (**Figures 1, 3, 4**) (Jiang et al., 2017). Whereas OBP representatives from subfamily II-A (**Figure 4**) together with OBP2 (**Supplementary Figure S3**) were found in the majority of this sensillum type, the atypical OBP subtype OBP12 from subfamily IV-A was present in a subpopulation of sensilla coeloconica. This observation seems to coincide with a previous finding that apart from a receptive role for leaf odors and organic acids (Ochieng and Hansson, 1999), a subset of sensilla coeloconica in locusts appears to be responsive to hygro- or thermo- stimuli (Altner et al., 1981). Such a functional versatility of this sensillum type may be based on distinct sets of cells equipped with specific receptors in combination with appropriate co-partners, e.g., OBP12. Remarkably, the atypical OBP subtype OBP12 belongs to the OBP gene family OBP59a, which is conserved in many insect species, except in Hymenoptera (Vieira and Rozas, 2011). For *Drosophila melanogaster* it has recently been shown that OBP59a is specifically expressed in sensilla coeloconica (Larter et al., 2016), similar to its counterpart in the desert locust (**Figure 4**).

An unexpected finding of this study is the expression of OBP2 in two types of sensilla, sensilla coeloconica and sensilla chaetica (**Figures 1, 3**). The two types of sensilla differ markedly in their external morphology and their functional implications(Montell, 2009; Rytz et al., 2013; Joseph and Carlson, 2015; Scott, 2018). On the other hand, in both sensillum types some common chemosensory genes are expressed, most notably the ionotropic receptor type IR25a, one of the co-receptors of divergent IRs (Abuin et al., 2011; Guo et al., 2013). Exploring the functional mode of IR25a in *Drosophila melanogaster* has recently uncovered a multidimensional role for this receptor type (Rimal and Lee, 2018) and it is conceivable that such a versatile function may also be assigned to the OBPs. In fact, it has been proposed that OBPs may be involved in quite different functions (Pelosi et al., 2006, 2014, 2017). In this regard, the observation that OBP2 is always accompanied by a set of other OBP subtypes in a sensillum (**Figures 2, 3**) may indicate that OBP2 operates in concert with other OBPs to fulfill the distinct functions conferred to the two types of sensilla.

One of the novel finding of this study was the discovery that the plus-C type-B subtype OBP9 is associated with the four antennal sensillum types. Although the functional implication of such a broad sensillum-association is unknown, one could imagine that OBP9, as an ubiquitous OBP, may contribute a general component for the interplay of co-localized OBP partners. Indeed, an interaction of OBP subtypes has been documented in mosquito species and the OBP complex showed a broader ligand spectrum (Qiao et al., 2011). This aspect may be of particular interest in view of the finding that in locust sensilla basiconica, with a large set of OR subtypes (Wang et al., 2015; Pregitzer et al., 2017), only a small set of OBPs is expressed (**Figure 7**). However, it can also not be excluded that OBP9 may be involved in quite different functions. In this context, it is interesting to note that in cockroach and honeybee, the chemosensory proteins, another important class of small soluble proteins, are involved in regulating tissue regeneration and embryonic development (Nomura et al., 1992; Maleszka et al., 2007; Cheng et al., 2015). Given such a broad sensillum-association, OBP9 may be involved in some general processes, such as development and/or survival of the auxiliary cells.

## Author Contributions

HB, JK, XJ, and PP: current study conception. XJ and MR: experiments conduction and the data acquisition. HB, JK, XJ, and PP: results interpretation. XJ and PP: preliminary manuscript composition. HB and JK: refinement and approval of final manuscript.

## Conflict of Interest Statement

The authors declare that the research was conducted in the absence of any commercial or financial relationships that could be construed as a potential conflict of interest.

## Supplementary Material

FIGURE S1 Classification of different subfamilies of locust OBPs. The phylogenetic tree shown was adapted from a previous study analyzing phylogenetic relationship of OBP families from four locust species (Jiang et al., 2017). The branches colored in red, green, blue, and magenta represent the clade I, II, III, and IV, respectively. The classification of the subfamily I-A, II-A, and III-A was based on emergence of a higher bootstrap values on the inner divergent nodes, while other subfamilies were categorized by the emerging topologies. The subtypes belonging to desert locust OBPs were colored and denoted accordingly.

FIGURE S2 A subset of sensilla chaetica selectively express OBP4 but not OBP8. Cells expressing the respective genes were visualized by using antisense riboprobes specifically targeting OBP4 and OBP8 and by means of two-color FISH. The position of cell clusters visualized by the DIG-labeled OBP4 probe (red) was delineated by dashed lines and is indicated in the images showing the OBP8 labeling and the merge of red and green fluorescence channels, respectively. Notably, no OBP8 labeling was detected. The interface between the cuticle and cellular layer is depicted by a white dashed line. Ch, sensilla chaetica; Ba, sensilla basiconica. Scale bar, 20 μm.

FIGURE S3 OBP2 and OBP12 are expressed in different cells in sensilla coeloconica (co). Specific antisense riboprobes against OBP2 and OBP12 were used to visualize the expressing cells by means of two-color FISH. The interface between the cuticle and the cellular layer is depicted by a white dashed line. Scale bar, 20 μm.
