# Supplementary figures and images for: Odorant Binding Proteins of the Desert Locust Schistocerca gregaria (Orthoptera, Acrididae): Topographic Expression Patterns in the Antennae
Source: Front Physiol. 2018 Apr 17;9:417. doi: 10.3389/fphys.2018.00417 (PMC5913285; doi:10.3389/fphys.2018.00417)

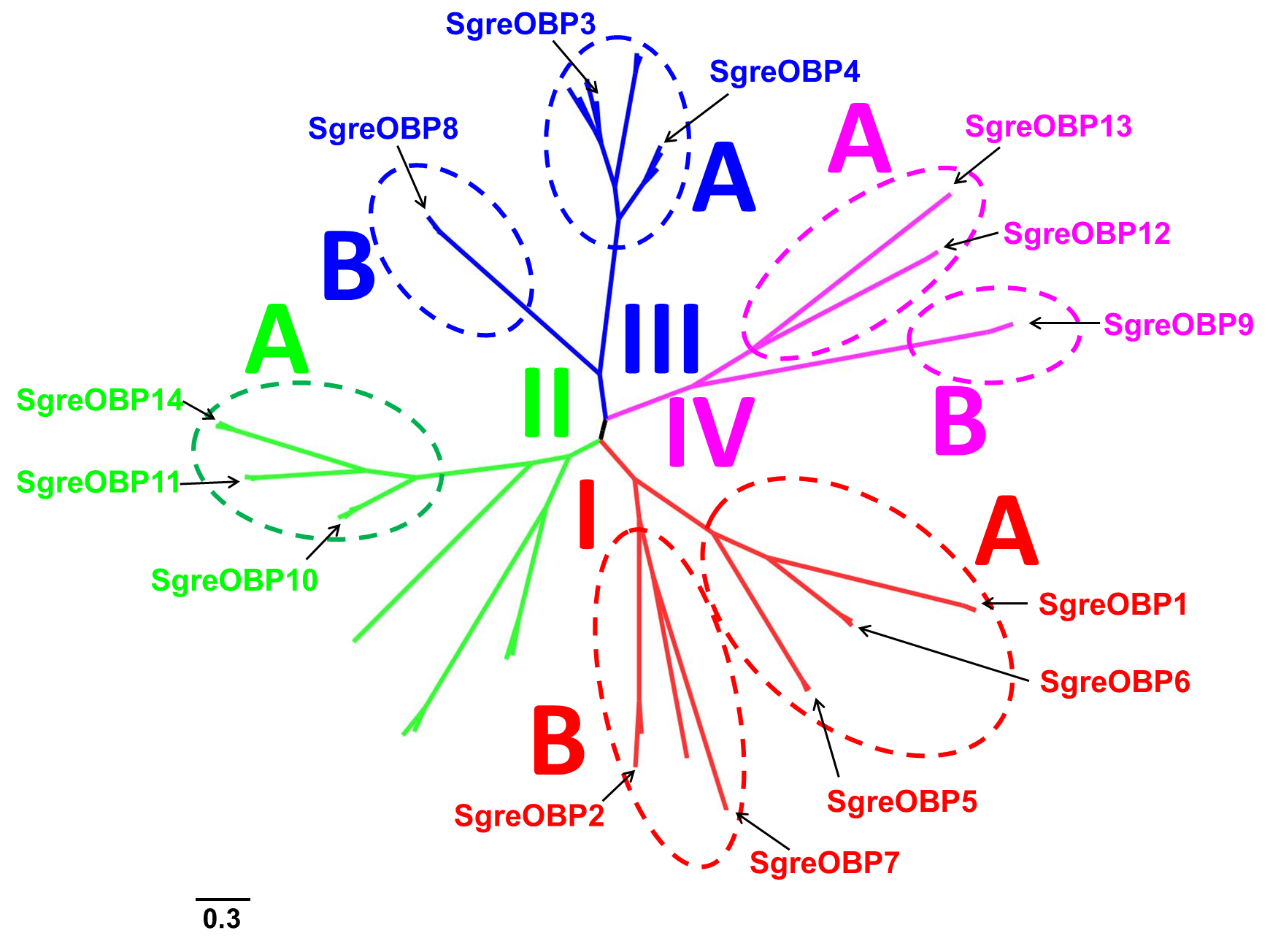

Supplement: FIGURE S1 — Classification of different subfamilies of locust OBPs. The phylogenetic tree shown was adapted from a previous study analyzing phylogenetic relationship of OBP families from four locust species (Jiang et al., 2017). The branches colored in red, green, blue, and magenta represent the clade I, II, III, and IV, respectively. The classification of the subfamily I-A, II-A, and III-A was based on emergence of a higher bootstrap values on the inner divergent nodes, while other subfamilies were categorized by the emerging topologies. The subtypes belonging to desert locust OBPs were colored and denoted accordingly. [file Image_1.TIF]

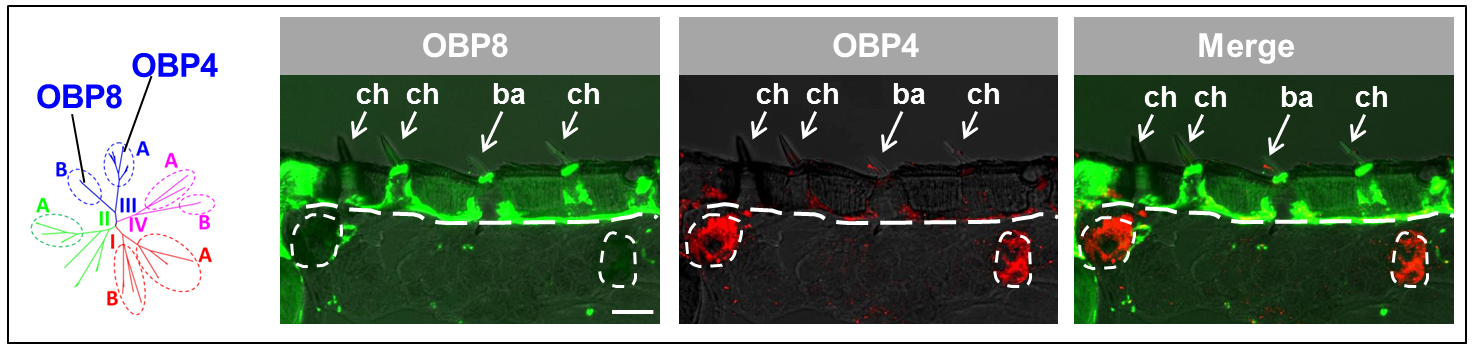

Supplement: FIGURE S2 — A subset of sensilla chaetica selectively express OBP4 but not OBP8. Cells expressing the respective genes were visualized by using antisense riboprobes specifically targeting OBP4 and OBP8 and by means of two-color FISH. The position of cell clusters visualized by the DIG-labeled OBP4 probe (red) was delineated by dashed lines and is indicated in the images showing the OBP8 labeling and the merge of red and green fluorescence channels, respectively. Notably, no OBP8 labeling was detected. The interface between the cuticle and cellular layer is depicted by a white dashed line. Ch, sensilla chaetica; Ba, sensilla basiconica. Scale bar, 20 μm. [file Image_2.TIF]

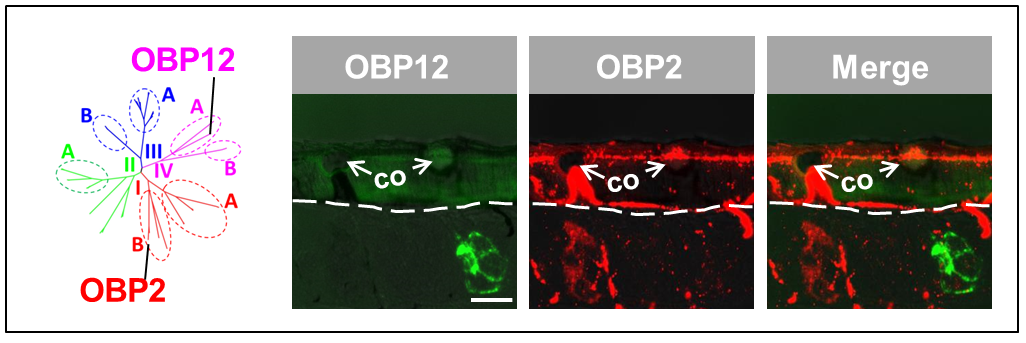

Supplement: FIGURE S3 — OBP2 and OBP12 are expressed in different cells in sensilla coeloconica (co). Specific antisense riboprobes against OBP2 and OBP12 were used to visualize the expressing cells by means of two-color FISH. The interface between the cuticle and the cellular layer is depicted by a white dashed line. Scale bar, 20 μm. [file Image_3.TIF]
